# Effectiveness and toxicity of conventional radiotherapy treatment for painful spinal metastases: a detailed course of side effects after opposing fields versus a single posterior field technique

**DOI:** 10.1007/s13566-017-0328-1

**Published:** 2017-09-19

**Authors:** Paulien G. Westhoff, Alexander de Graeff, Evelyn M. Monninkhof, Ilse de Pree, Marco van Vulpen, Jan Willem H. Leer, Corrie A. M. Marijnen, Yvette M. van der Linden, The Dutch Bone Metastasis Study Group

**Affiliations:** 1 Department of Radiation Oncology, University Medical Center Utrecht, PO Box 85500, 3508 GA Utrecht, the Netherlands; 2 Department of Radiation Oncology, Radboud University Medical Center, PO Box 9101, 6500 HB Nijmegen, the Netherlands; 3 Department of Medical Oncology, University Medical Center Utrecht, PO Box 85500, 3508 GA Utrecht, the Netherlands; 4 Julius center for Health Sciences and Primary Care, University Medical Center Utrecht, PO Box 85500, 3508 GA Utrecht, the Netherlands; 5 Department of Radiation Oncology, Erasmus Medical Center, PO Box 5201, 3008 AE Rotterdam, the Netherlands; 6 Department of Radiation Oncology, Leiden University Medical Center, PO Box 9600, 2300 RC Leiden, the Netherlands

**Keywords:** Palliative radiotherapy, Spinal metastases, Side effects, Toxicity, Bone metastases

## Abstract

**Background:**

Conventional radiotherapy for painful spinal metastases can be delivered with a single posterior-anterior (PA) or two opposed anterior-posterior (APPA) fields. We studied the effectiveness and toxicity of both techniques and studied whether treatment technique was predictive for abdominal and skin toxicity.

**Patients and methods:**

Within the Dutch Bone Metastasis Study, 343 patients received 8 Gray in a single fraction or 24 Gray in six fractions for painful spinal metastases. Treatment technique was not randomized. At baseline and weekly during follow-up, patients reported pain and other physical complaints. Any complaint increasing within 4 weeks after treatment was noted as a side effect. Pain response was calculated according to international standards, taking into account changes in pain score and medication. Repeated measurement analyses and multivariate logistic analyses were performed.

**Results:**

Patients were mainly treated on the thoracic (34%) and lumbar (53%) spine and 73% received a PA field. Pain response was similar between both techniques (74%). In patients treated at the thoraco-lumbar and lumbar spine, with multiple fractions, significantly more abdominal complaints were noticed. In multivariate analysis, radiotherapy technique did not predict for side effects.

**Conclusion:**

Conventional radiotherapy of painful spinal metastases provides limited toxicity. Radiotherapy technique is not an independent predictor of abdominal and skin toxicity of irradiation.

## Introduction

For patients with painful bone metastases, radiotherapy is an effective treatment, with a pain response rate of more than 60%. The golden standard is to treat these patients with a single fraction of 8 Gray (Gy) [1–3], aiming at pain relief with minimal toxicity.

In general, side effects from this treatment are mild and depend on factors like dose, field size, and the anatomic area being irradiated [1, 4–6]. In several studies in patients treated with radiotherapy for painful bone metastases, toxicity rates between 35 and 46% are reported, consisting mainly of nausea and/or vomiting [7–9]. A recent study in 32 patients treated for painful bone metastases showed that over 50% of patients had complaints of nausea and/or vomiting, despite receiving prophylactic anti-emetic treatment [10].

Radiotherapy to spinal metastases can be delivered with different treatment techniques. Highly conformal treatment techniques like intensity-modulated radiotherapy (IMRT) and volumetric arc radiotherapy (VMAT) are used more and more, often to higher doses. Still, frequently used conventional techniques are a single posterior-anterior (PA) field or two parallel opposed fields from anterior and posterior (APPA). The advantage of a PA field is the sparing of anterior organs like the bowel, although the coverage of the vertebrae might be suboptimal [11]. By using a PA field, the dose to the posterior skin and paraspinal musculature can be high, which can be a problem for future surgical interventions [12–14]. The advantage of the APPA technique is a better coverage of the vertebrae [11], while sparing the posterior skin and paraspinal musculature. A disadvantage is the higher dose in the anterior organs, possibly leading to more abdominal side effects. In the literature, however, no prospective data on the toxicity of both techniques have been published.

The aim of the present analysis is to study the differences in effectiveness and toxicity of PA and APPA techniques for the irradiation of painful spinal metastases and to identify factors predictive for side effects of treatment. We studied patients who received radiotherapy for painful spinal metastases within the randomized Dutch Bone Metastasis Study (DBMS) [1].

## Patients and methods

Details of the patient population and study protocol of the DBMS were published elsewhere [1, 15]. In summary, the DBMS was a nationwide, randomized trial in patients with uncomplicated painful bone metastases. Between 1996 and 1998, a total of 1157 patients with painful bone metastases were randomized between a single fraction (SF) of 8 or 24 Gy in six fractions. The study showed equal effectiveness of a SF versus multiple fractions (MF) with regard to pain response, which was the primary endpoint. All patients provided informed consent and the medical ethics committees of participating institutions approved the study.

### Patients

Patients with metastases in the cervical spine were excluded from the DBMS [1, 15]. In total, 348 patients were treated for painful metastases in the thoracic, lumbar, or sacral spine. Data on spinal location and treatment technique were available in 343 patients (99%). For spinal location, the treated level of the spine (i.e., thoracic, lumbar, or sacral) was registered, without specification of the specific vertebra or vertebrae irradiated. Treatment was performed with conventional treatment techniques, using either a PA field or APPA fields contributing each 50% of the total dose; no other techniques were used. The prescription depth for PA fields was typically at 4 to 6 cm, depending on the depth of the vertebra. The choice for treatment technique was left to the decision of the treating radiation oncologist and was mostly dependent on institutional policy.

### Questionnaires

At randomization and during follow-up, patients filled out 13 weekly questionnaires and monthly afterwards until 2 years of follow-up, death, or closure of the study in December 1998. The questionnaires consisted, among others, of the Rotterdam Symptom Checklist (RSCL) [16], a pain scale, pain medication intake, and questions about itching and painful skin. No data were available on the use of anti-emetics or anti-diarrhea medication. The following items were studied to determine toxicity: diarrhea, abdominal pain, nausea, and vomiting. These scores were grouped into the variable “abdominal complaints.” Itching and painful skin were grouped into the variable “skin complaints.” All items were rated on a 4-point Likert-type scale, ranging from 1 (no complaints) to 4 (severe complaints). To facilitate interpretation, all sum scores were standardized to the range of 0 (no complaints) to 10 (severe complaints). Besides sum scores, the individual item scores were also studied. As radiotherapy of the lower spine is more likely to affect the bowel, we studied the individual abdominal items for the treated thoraco-lumbar, lumbar, and lumbo-sacral vertebrae separately. Pain was measured using an 11-point numeric rating scale, ranging from 0 (no pain) to 10 (the worst pain imaginable). A pain score of at least 2 was required to enter the study [1].

### Statistical analyses

Pain response was calculated according to international criteria, taking into account changes in pain medication and pain score [17]. No fixed time interval from the date of randomization was applied. A response was calculated if at least two successive follow-up pain scores were available, which was possible in 325 patients (95%).

To compare the categorical variables at baseline, chi-square tests were used. To visualize and compare the course of side effects over time, we used repeated measurement analyses (mixed procedure), a longitudinal data analysis technique. Analyses were also performed adjusted for treatment institute to take into account potential confounding by indication by institutional choice for treatment technique. *p* values are based on two-sided tests and considered significant if *p* < 0.05. Figures were created based on the least square means of the repeated measurements.

To assess which baseline variables were predictive for toxicity, the complaints variable was dichotomized into having or not having complaints. For that purpose, we compared the maximal complaint scores 1 to 4 weeks after treatment with the baseline scores. If a score was higher than the baseline score, the patient was considered as having side effects of radiotherapy. The time period of 4 weeks was chosen because by then most side effects would be present.

We applied multivariate logistic regression analyses to relate candidate predictors to toxicity. First, we started with a full model, including all preselected variables. Subsequently, we eliminated the variables by a backward selection process with a threshold *p* value of 0.20, based on likelihood-ratio test results. The chosen *p* value of 0.20 intends to limit the loss of information and to select also weaker predictors, although at the cost of including “noise” variables [18]. The preselected baseline variables, based on the literature and clinical experience, were primary tumor (breast, prostate, lung, or other cancer), age (≤ 65 years or > 65 years), gender (male or female), Karnofsky performance status (KPS) [19] (≤ 60, 70–80, or 90–100), pain score (2–4, 5–7, or 8–10), presence of visceral metastases (yes or no), concomitant systemic therapy (yes or no), treatment arm (1 × 8 or 6 × 4 Gy), opioids (yes or no), spinal localization (thoracic, thoraco-lumbar, lumbar, or lumbo-sacral spine), and treatment technique (PA or APPA).

The database was analyzed using IBM SPSS statistics for Windows version 20.0 (IBM Corp., Armonk, NY, USA) and SAS software (version 9.2, SAS Institute Inc., Cary, NC, USA).

## Results

### Baseline characteristics

In general, patients with spinal metastases did not differ from the entire population of 1157 patients with bone metastases. Table 1 shows baseline characteristics of the study population (*n*, 343). Primary tumors were mainly breast (42%), prostate (24%), and lung (20%) cancer. The mean age was 65 years (range 32–89 years) and 52% was male. The majority of patients was in good to moderate condition; 71% had a KPS of 70 or higher. The mean pain score at baseline was 6.4 (range 2–10). Visceral metastases were documented in 28% of patients; 55% of patients received concomitant systemic therapy at the time of randomization.

**Table 1:** Baseline characteristics of patients with painful spinal metastases, treated with a PA or an APPA technique

	*n*	Entire cohort	Spinal patients	PA	APPA	Difference PA versus APPA^a^
		1157	343	250	93	
Primary tumor						n.s.
Breast cancer		451 (39%)	145 (42%)	110 (44%)	35 (38%)	
Prostate cancer		267 (23%)	83 (24%)	63 (25%)	20 (22%)	
Lung cancer		287 (25%)	68 (20%)	48 (19%)	20 (22%)	
Other		152 (13%)	47 (14%)	29 (12%)	18 (19%)	
Age						n.s.
≤ 65 years		565 (49%)	167 (49%)	120 (48%)	47 (51%)	
> 65 years		592 (51%)	176 (51%)	130 (52%)	46 (50%)	
Gender						n.s.
Male		624 (54%)	178 (52%)	125 (50%)	53 (57%)	
Female		533 (46%)	165 (48%)	125 (50%)	40 (43%)	
KPS						n.s.
90–100		221 (19%)	67 (20%)	47 (19%)	20 (22%)	
70–80		587 (51%)	176 (51%)	132 (53%)	44 (47%)	
20–60		343 (30%)	100 (29%)	71 (28%)	29 (31%)	
Pain score						n.s.
2–4		234 (20%)	71 (21%)	54 (22%)	17 (18%)	
5–7		550 (48%)	155 (45%)	116 (46%)	39 (42%)	
8–10		366 (32%)	117 (34%)	80 (32%)	37 (40%)	
Visceral metastases						n.s.
No		838 (72%)	247 (72%)	178 (71%)	69 (74%)	
Yes		319 (28%)	96 (28%)	72 (29%)	24 (26%)	
Systemic therapy						0.009
No		531 (46%)	156 (46%)	103 (41%)	53 (57%)	
Yes		626 (54%)	187 (55%)	147 (59%)	40 (43%)	
Treatment schedule						n.s.
1 × 8 Gy		578 (50%)	171 (50%)	129 (52%)	42 (45%)	
6 × 4 Gy		579 (50%)	172 (50%)	121 (48%)	51 (55%)	
Pain medication						n.s.
No opioids		667 (58%)	170 (50%)	123 (49%)	47 (51%)	
Opioids		490 (42%)	173 (50%)	127 (51%)	46 (50%)	
Spinal localization						n.s.
Thoracic spine			117 (34%)	90 (36%)	27 (29%)	
Thoraco-lumbar spine			32 (9%)	27 (11%)	5 (5%)	
Lumbar spine			183 (53%)	124 (50%)	59 (63%)	
Lumbo-sacral spine			11 (3%)	9 (4%)	2 (2%)	

*PA,* Posterior-anterior field; *APPA,* Anterior-posterior and posterior-anterior field; *KPS,* Karnofsky performance score; *Gy,* Gray; *n.s.,* not significant

^a^Chi-square

Two hundred fifty patients (73%) were treated with a single PA field and 93 patients (27%) with an APPA technique. The most frequently treated localization was the lumbar spine (53%), followed by the thoracic spine (34%). The remaining patients were treated at overlapping regions. Baseline characteristics did not differ between both treatment technique groups, except for systemic therapy. More patients in the group treated with a PA field were treated with systemic therapy (59%), compared to patients treated with an APPA technique (43%, *p* = 0.009).

At baseline, the mean scores of the individual complaints items were low, varying from 1 (no complaints) to 2 (minor complaints), on a scale from 1 to 4. The mean sum score of abdominal complaints was 1.3 (range 0–7.5, on a scale from 0 to 10) and the mean sum score of skin complaints was 0.7 (range 0–8.3, on a scale from 0 to 10). No baseline differences in items or sum scores between the two treatment groups were observed.

A preference per treatment institute was noticed for treatment technique. Institute policy and preferences mainly determined the choice of technique, instead of individual patient characteristics.

### Pain response

In total, 241 (74%) of the 325 evaluable patients had a pain response to radiotherapy, with no significant difference between the two treatment techniques (74% each). The pain response rate is comparable to that of the entire DBMS population.

### Side effects

**Fig. 1:**
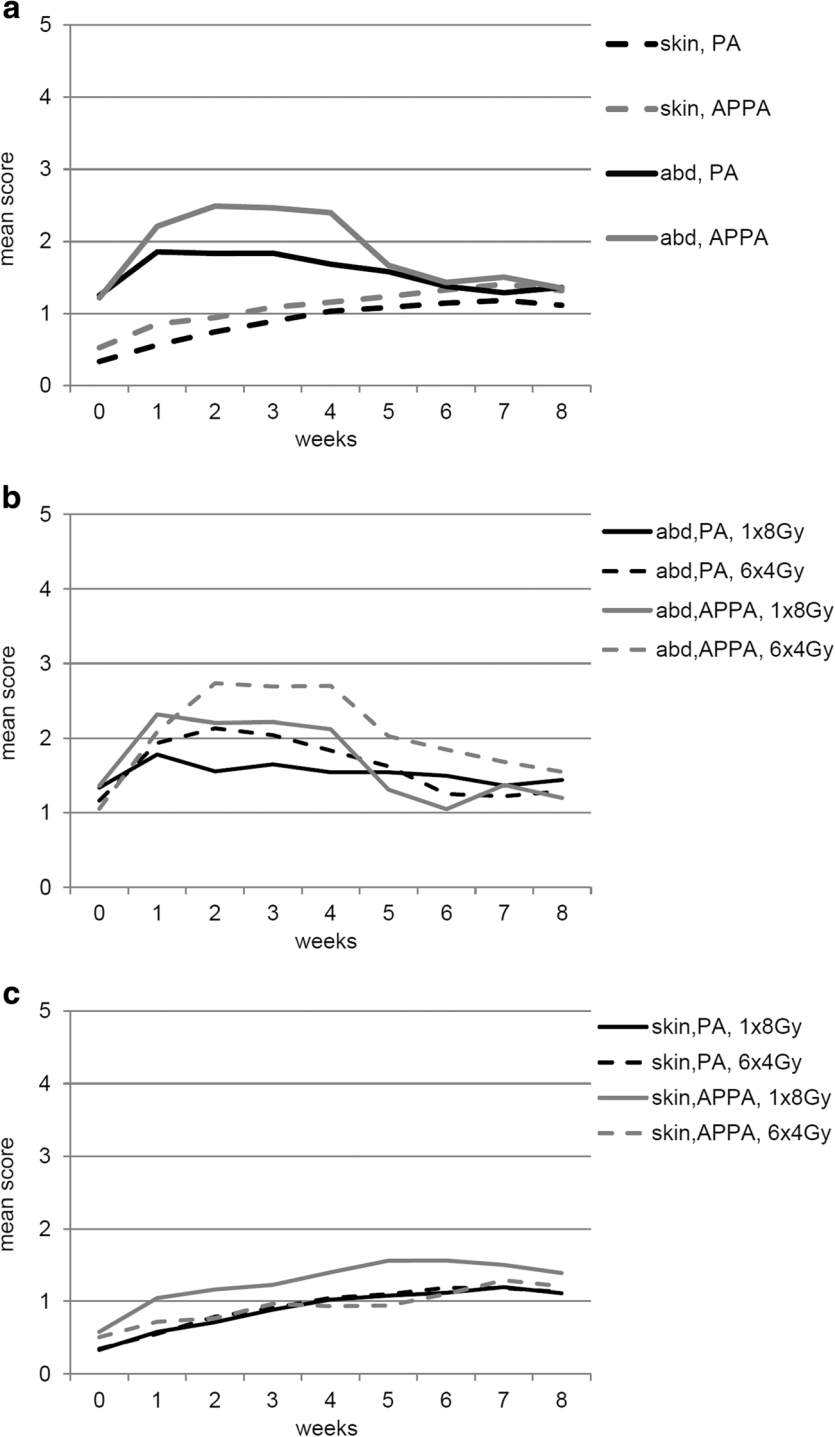
The course of complaints (range of score 0 to 10) after radiotherapy for painful spinal metastases. **a** Abdominal and skin complaints per treatment technique. **b** Abdominal complaints per treatment technique and fractionation schedule. **c** Skin complaints per treatment technique and fractionation schedule

Side effects were minor. In general, patients experienced more abdominal complaints than skin complaints. Four and 8 weeks after treatment, respectively, 264 (77%) and 229 (67%) patients returned questionnaires. Figure 1 shows the course of complaints in the first weeks after treatment. Patients treated with an APPA technique experienced more abdominal complaints compared to patients treated with a PA field. This difference was temporary; abdominal complaints were comparable 5 weeks after treatment. Skin complaints increased minimally over time, irrespective of treatment technique (Fig. 1a). For both techniques, patients receiving multiple fractions experienced more abdominal complaints than patients receiving a single fraction (Fig. 1b). Differences in skin complaints were hardly noticed (Fig. 1c).

**Fig. 2:**
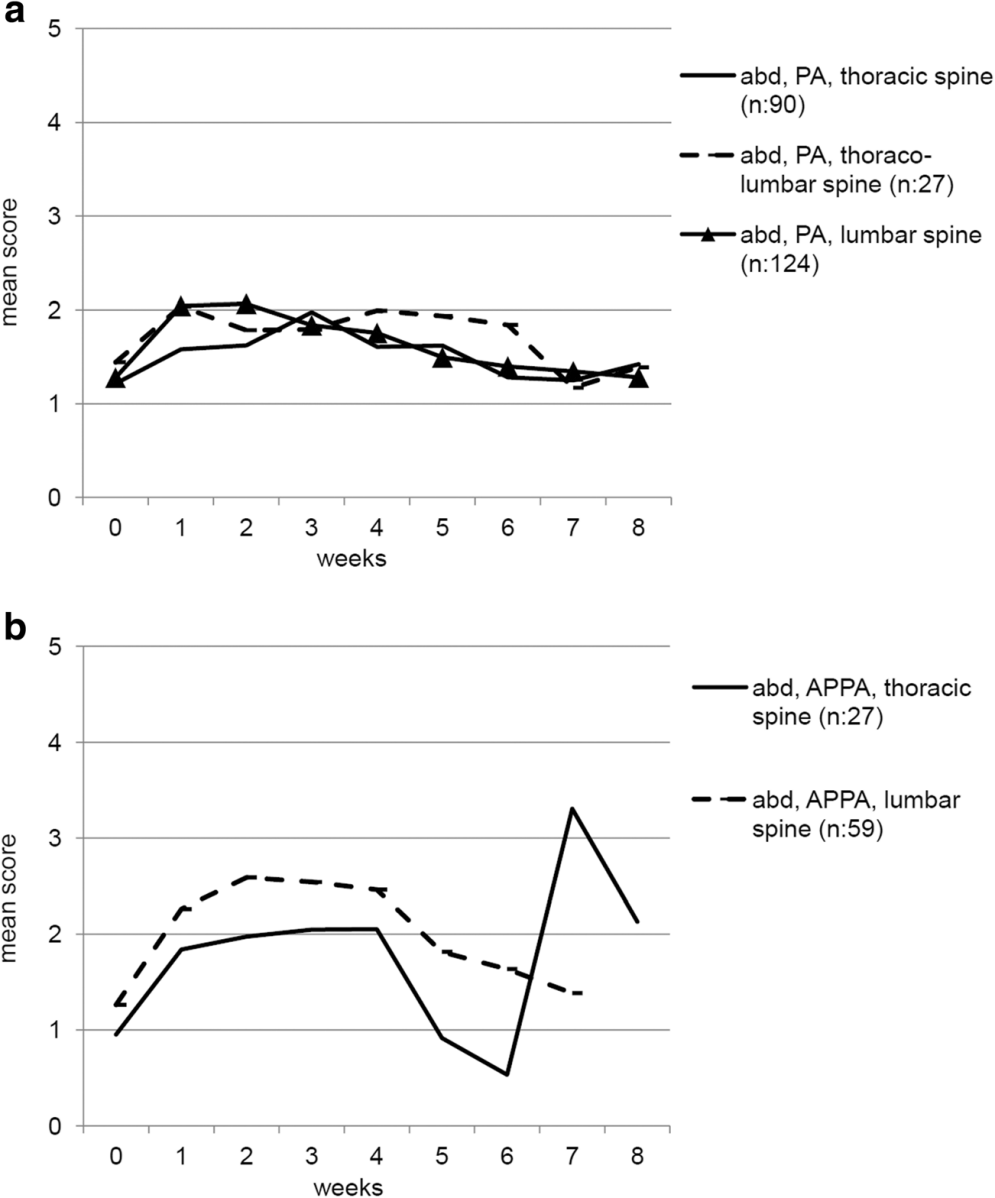
The course of abdominal complaints (range of score 0 to 10) after radiotherapy for painful spinal metastases. To facilitate interpretation, subgroups with less than 10 patients were not shown in the figure (lumbo-sacral spine PA (*n*, 9), thoraco-lumbar spine APPA (*n*, 5), lumbo-sacral spine APPA (*n*, 2)). **a** Abdominal complaints using the PA technique per location. **b** Abdominal complaints using the APPA technique per location

In patients treated with both techniques, the course of complaints was similar for all anatomical localizations, although most outspoken for the lumbar spine in patients treated with an APPA technique (Fig. 2). For skin complaints, only the two patients treated at the lumbo-sacral spine with an APPA technique showed a distinctive increase, to a maximum mean score of 3.4 (scale of 0–10).

Studying the separate side effects for all 343 patients, a trend was noticed towards more vomiting and abdominal pain in patients treated with the APPA technique (*p* = 0.054 and *p* = 0.053, respectively). Patients treated with the APPA technique had significantly more severe complaints of diarrhea (*p* = 0.044). No significant difference was noticed for nausea. Studying the abdominal side effects for the lower spine (all patients, excluding those treated on the thoracic spine only), there were statistically significant differences between treatment techniques. For all studied items (nausea, vomiting, abdominal pain, and diarrhea), patients treated with the APPA technique experienced more complaints than patients treated with the PA technique (*p* values all <0.009).

Table 2 shows the results of the multivariate analyses. Treatment schedule and location were independent predictors for abdominal complaints. Patients treated with a single fraction had a lower risk of abdominal complaints (OR 0.49 (95% CI 0.29–0.81)) compared to multiple fractions. Patients treated at the thoraco-lumbar and lumbar spine had a higher risk of abdominal complaints (OR 2.51 (0.93–6.80) and 2.29 (1.34–3.93), respectively), compared to radiotherapy of the thoracic spine. For skin complaints (Table 3), primary tumor and localization were predictive in multivariate analyses. Patients with lung cancer (OR 2.27 (1.20–4.30) compared to breast cancer) had a higher risk of skin complaints. Patients treated at the lumbo-sacral spine (OR 1.83 (0.52–6.49) compared to radiotherapy of the thoracic spine) had a higher risk, while patients treated at the lumbar spine had a lower risk of skin complaints (OR 0.54 (0.32–0.91)) compared to the thoracic spine.

**Table 2:** Analysis of potential predictors for developing abdominal complaints within 4 weeks after treatment for painful spinal metastases

Baseline variables	% of patients with	Odds ratio (95% CI)	
	Abdominal complaints	UVA^a^	MVA^a^
Primary tumor			
Breast cancer	73%	1.00	#
Prostate cancer	71%	0.90 (0.49–1.67)	
Lung cancer	69%	0.84 (0.43–1.64)	
Other	64%	0.68 (0.33–1.42)	
Age			
≤ 65 years	70%	1.00	#
> 65 years	70%	1.01 (0.62–1.64)	
Gender			
Male	71%	1.00	#
Female	70%	0.95 (0.59–1.54)	
KPS			
90–100	65%	1.00	#
70–80	73%	1.16 (0.61–2.20)	
20–60	70%	0.78 (0.39–1.55)	
Pain score			
2–4	69%	1.00	#
5–7	73%	1.22 (0.65–2.30)	
8–10	68%	0.96 (0.49–1.86)	
Visceral metastases			
No	69%	1.00	#
Yes	74%	1.26 (0.72–2.22)	
Systemic therapy			
No	73%	1.00	#
Yes	68%	0.78 (0.48–1.27)	
Treatment schedule			
6 × 4 Gy	78%	1.00	1.00
1 × 8 Gy	63%	0.49 (0.30–0.81)	0.49 (0.29–0.81)
Pain medication			
No opioids	71%	1.00	#
Opioids	70%	0.95 (0.59–1.54)	
Spinal localization			
Thoracic spine	60%	1.00	1.00
Thoraco-lumbar spine	79%	2.44 (0.91–6.54)	2.51 (0.93–6.80)
Lumbar spine	77%	2.22 (1.31–3.77)	2.29 (1.34–3.93)
Lumbo-sacral spine	45%	0.56 (0.16–1.94)	0.65 (0.18–2.30)
Treatment technique			
PA	69%	1.00	#
APPA	75%	1.35 (0.76–2.37)	

*95%, CI* 95% confidence interval; *UVA,* Univariate analysis; *MVA,* Multivariable analysis; *KPS,* Karnofsky performance status; *PA,* Posterior-anterior field; *APPA,* Anterior-posterior and posterior-anterior field

^a^Logistic regression analysis

#Did not remain in the final model

**Table 3:** Analysis of potential predictors for developing skin complaints within 4 weeks after treatment for painful spinal metastases

Baseline variables	% of patients with	Odds ratio (95% CI)	
	Skin complaints	UVA^a^	MVA^a^
Primary tumor			
Breast cancer	31%	1.00	1.00
Prostate cancer	28%	0.87 (0.47–1.61)	0.95 (0.50–1.79)
Lung cancer	49%	2.19 (1.17–4.11)	2.27 (1.20–4.30)
Other	36%	1.26 (0.61–2.62)	1.28 (0.61–2.70)
Age			
≤ 65 years	39%	1.00	#
> 65 years	30%	0.66 (0.41–1.06)	
Gender			
Male	35%	1.00	#
Female	33%	0.92 (0.58–1.47)	
KPS			
90–100	34%	1.00	#
70–80	31%	0.84 (0.46–1.56)	
20–60	40%	1.29 (0.66–2.51)	
Pain score			
2–4	29%	1.00	#
5–7	34%	1.22 (0.65–2.31)	
8–10	38%	1.48 (0.76–2.90)	
Visceral metastases			
No	33%	1.00	#
Yes	38%	1.27 (0.76–2.14)	
Systemic therapy			
No	38%	1.00	#
Yes	31%	0.75 (0.47–1.20)	
Treatment schedule			
6 × 4 Gy	31%	1.00	#
1 × 8 Gy	37%	1.34 (0.84–2.15)	
Pain medication			
No opioids	33%	1.00	#
Opioids	35%	1.09 (0.68–1.75)	
Spinal localization			
Thoracic spine	42%	1.00	1.00
Thoraco-lumbar spine	39%	0.91 (0.39–2.14)	0.95 (0.40–2.25)
Lumbar spine	27%	0.52 (0.31–0.88)	0.54 (0.32–0.91)
Lumbo-sacral spine	55%	1.69 (0.49–5.89)	1.83 (0.52–6.49)
Treatment technique			
PA	33%	1.00	#
APPA	37%	1.19 (0.70–2.02)	

*95%, CI* 95% confidence interval; *UV, A* univariate analysis; *MVA,* Multivariable analysis; *KPS,* Karnofsky performance status; *PA,* Posterior-anterior field; *APPA,* Anterior-posterior and posterior-anterior field

^a^Logistic regression analysis

#Did not remain in the final model

Treatment technique did not predict for abdominal or skin toxicity after radiotherapy. When studying patients per treatment arm, treatment technique was not significantly associated with abdominal or skin toxicity.

## Discussion

This study showed that treatment technique did not predict for abdominal nor skin complaints. Pain response rates did not differ between both treatment techniques. In a multivariate model, fractionation schedule and treated localization were independent predictors of abdominal complaints. Primary tumor and treated location appeared to be predictors of skin complaints.

Although nowadays more conformal techniques and even stereotactic radiotherapy are frequently used in patients with painful bone metastases, the benefits of those techniques compared to the conventional techniques still remain to be proven. All available data show that a dose higher than 8 Gy is not superior to a single dose of 8 Gy in terms of pain control [20]. These techniques have the disadvantage that they are more time consuming in terms of preparation, additional imaging modalities (such as magnetic resonance imaging) needed, and a more complex and technically demanding treatment planning [21–23]. Furthermore, they are more expensive than conventional treatment techniques [24] and treatment time is in general prolonged, which causes more inconvenience for the patients. Therefore, for the majority of patients, conventional techniques still remain the treatment of first choice [25].

In general, the reported side effect scores were relatively low. This does not imply that those side effects are not relevant, since even mild complaints might be burdensome. In a study among 368 patients receiving radiotherapy, patients with nausea, although with mild severity in 72% of patients, had a lower QoL and a lower overall level of well-being than patients without nausea [26].

Our results showing more abdominal complaints with the multiple fraction treatment are in line with the results from Chow et al. in re-irradiated patients with painful bone metastases [27]. They described more vomiting, loss of appetite, and diarrhea after 20 Gy in multiple fractions compared to a single fraction of 8 Gy. They also described more redness of the skin after multiple fractions, which was not noticed in our analyses, although redness was not specifically questioned.

The abdominal side effects of the APPA technique were more prominent in patients treated on the lower spine. In this part of the spine, the vertebrae are located relatively ventral. An APPA technique gives a better dose coverage, due to the deep location of the target volume, with the anterior body of the fifth lumbar vertebra located at a mean depth of 12 cm [11]. A PA technique might lead to a lower dose on the ventral part of the vertebral body, which can be a disadvantage, since previous studies have shown that a dose of 8 Gy results in a higher pain response rate than lower doses [28, 29]. On the other hand, in this study population, the response rate does not differ between treatment techniques.

Another disadvantage of the PA technique at the lower spine might be the high skin dose when trying to cover the ventral part of the vertebral body [11]. We did not notice skin side effects related to treatment technique, but this might be due to the type of questions asked and the lack of an objective physical examination. An option could be to treat this location with a three-field or intensity-modulated technique, thereby avoiding bowel structures [30] and the skin [31, 32]. However, these conformal techniques are more time consuming, for patients and logistics [33], and not available in every institution. A more conformal, but efficient and easy technique is a single PA field using 10MV, with the addition of a second AP field, contributing less than 50% of the dose, to increase the dose ventrally to at least 85% of the prescribed dose. In this way, side effects to the bowel can be minimized.

In our multivariate analyses, we found that patients with bone metastases from lung cancer are at increased risk of skin complaints. We have no reason to believe that those patients are more sensitive to radiotherapy. We also found that patients treated at the lumbo-sacral spine have more skin complaints. An explanation might be the varying depth of lumbal and sacral vertebra [11], possibly leading to more skin dose when trying to cover the entire vertebral bodies with a PA field. However, we believe those skin complaints to be of minor relevance, due to the limited increase in complaints.

A disadvantage of our analyses is, firstly, that we are not informed about the intake of anti-emetics and/or anti-diarrhea medication. It has been shown that the decision on prescribing medication differs per physician [34], so patients from some physicians might have had medication for side effects, while others had not. Secondly, the choice of treatment technique was not randomized, increasing the risk of confounding by indication. We did notice a preference per treatment institute. Since institute policy and preferences mainly determined the choice of technique, instead of individual patient characteristics, we also adjusted our analyses for treatment institute as sensitivity analyses, which showed similar outcomes. Thirdly, patients reported their complaints once a week. Perhaps if reported with smaller intervals, minor but relevant differences in toxicity would have been noted. Fourthly, only 93 patients were treated with the APPA technique, with a subgroup of 51 patients treated with six fractions of 4 Gy and 42 patients with a single fraction of 8 Gy. Finally, no data were known about dose distribution.

On the other hand, this dataset provides a unique insight in patients receiving palliative radiotherapy, due to the number of patients included and the frequency and contents of the prospective patient-reported follow-up. Although our data were collected from 1996 until 1998, we believe the results presented here are still representative for current patients receiving palliative radiotherapy for spinal metastases, which is still delivered mainly using AP and APPA fields. And, while improvements in systemic therapy have occurred over the last years, the most frequently applied treatment for painful bone metastases is palliative radiotherapy, with a single fraction of 8 Gy as the golden standard [2]. Although medication might help to prevent the reported side effects [35], we believe it is better to try to avoid any side effects by using an optimal treatment technique, especially in this patient group with frequent co-medication and/or systemic therapies.

In conclusion, we advocate the use of a single fraction to treat patients with painful uncomplicated spinal metastases. Based on our analysis, when conventional techniques are used, there is no preference for either a PA or an APPA technique. If higher total doses are needed, we advise to search for a more conformal treatment technique to avoid high doses to the abdomen, specifically when treating the lower spine.
